# Abiotic Stresses: General Defenses of Land Plants and Chances for Engineering Multistress Tolerance

**DOI:** 10.3389/fpls.2018.01771

**Published:** 2018-12-07

**Authors:** Mei He, Cheng-Qiang He, Nai-Zheng Ding

**Affiliations:** College of Life Science, Shandong Normal University, Jinan, China

**Keywords:** abiotic stresses, land plants, general defenses, regulatory network, multistress tolerance

## Abstract

Abiotic stresses, such as low or high temperature, deficient or excessive water, high salinity, heavy metals, and ultraviolet radiation, are hostile to plant growth and development, leading to great crop yield penalty worldwide. It is getting imperative to equip crops with multistress tolerance to relieve the pressure of environmental changes and to meet the demand of population growth, as different abiotic stresses usually arise together in the field. The feasibility is raised as land plants actually have established more generalized defenses against abiotic stresses, including the cuticle outside plants, together with unsaturated fatty acids, reactive species scavengers, molecular chaperones, and compatible solutes inside cells. In stress response, they are orchestrated by a complex regulatory network involving upstream signaling molecules including stress hormones, reactive oxygen species, gasotransmitters, polyamines, phytochromes, and calcium, as well as downstream gene regulation factors, particularly transcription factors. In this review, we aimed at presenting an overview of these defensive systems and the regulatory network, with an eye to their practical potential via genetic engineering and/or exogenous application.

## Introduction

Land plants are living in an inherently harsh environment ever since their emergence. A large variety of physical or chemical factors are hostile to them, including low or high temperature, deficient or excessive water, high salinity, heavy metals, and ultraviolet (UV) radiation, among others. These stresses, collectively referred to as abiotic stresses, are posing a severe threat to agriculture and the ecosystem, accounting for great crop yield loss (Wang et al., 2003; Wania et al., 2016). Salt stress is the most stubborn one magnified by ever-increasing salinization of arable land worldwide (Munns and Tester, 2008; Yuan et al., 2015b). Most plants cannot survive when NaCl concentrations exceed 200 mM (Flowers and Colmer, 2008; Zhou J.C. et al., 2016) because high salinity extensively impinges on their lifecycle comprising, if available, seed germination, seedling establishment, vegetative growth, and flower fertility (Guo et al., 2012, 2015, 2018), as a consequence of ionic toxicity, osmotic pressure, oxidative damage, and nutritional shortage (Zhao et al., 2010; Feng et al., 2014a,b). More seriously, it is interlinked with drought, another global issue, which can be aggravated by extreme temperatures (Ashraf and Foolad, 2007; Slama et al., 2015).

Due to their sessile nature, plants have to confront the stresses and develop potent adaptive tactics to avoid or tolerate their adverse effects so as to survive and to thrive. Plenty of cellular, physiological and morphological defenses have been established. The most apparent one is the cuticle, a universal outmost shield (Shepherd and Wynne Griffiths, 2006; Yeats and Rose, 2013; Fich et al., 2016). It is also impressive that recretohalophytes even evolved a specialized organ to excrete salt, as represented by the epidermal salt gland of *Limonium bicolor* (Yuan et al., 2013, 2016a). Tremendous progress has been made toward understanding the biochemical and molecular mechanisms underpinning the defenses, owing to forward and reverse genetic approaches as well as genome-wide analyses conducted on various model species like the classical model *Arabidopsis thaliana* and its extremophyte relative *Thellungiella salsuginea* that has exceptional multistress resistance (Amtmann, 2009; Wang J.S. et al., 2017).

**FIGURE 1:**
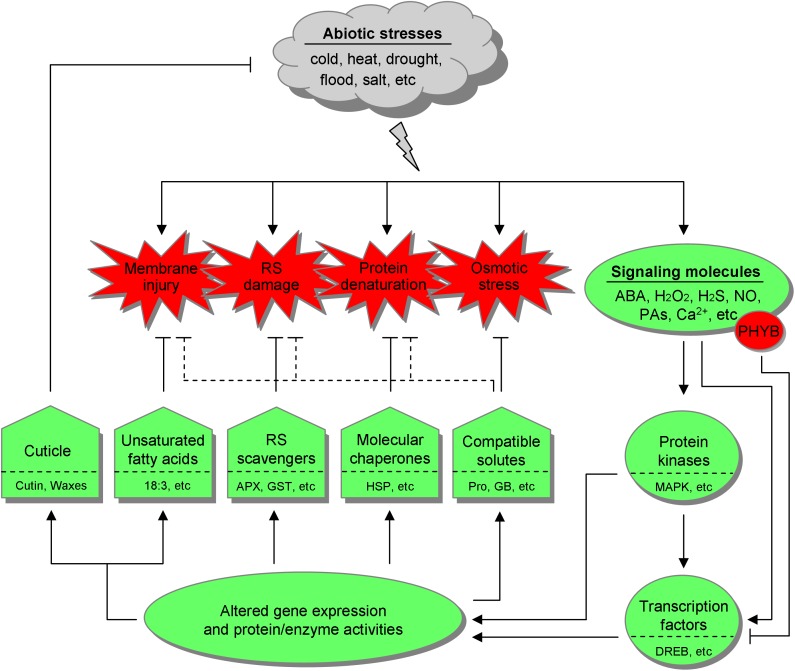
The general defense systems and the underlying regulatory network in botanic responses to abiotic stresses. Different abiotic stresses, such as cold, heat, drought, flood, and salt can provoke common cellular disorder and secondary stresses, including membrane injury, reactive species (RS) damage, protein denaturation, and osmotic stress, which are also interconnected with each other. Accordingly, land plants have resorted to unsaturated fatty acids, RS scavengers, molecular chaperones, and compatible solutes. Some compatible solutes may also be involved in counteracting other adverse effects, as indicated with dotted inhibitory lines. Besides, the cuticle serves as the universal outermost shield. Upon stress stimulation, signaling molecules mobilize the downstream effectors, primarily protein kinases and transcription factors, leading to altered gene expression and protein/enzyme activities, thereby launching the defense systems. Notably, phytochrome B (PHYB) is emerging as a negative regulator in stress tolerance. 18:3, linolenic acid; APX, ascorbate peroxidase; GST, glutathione *S*-transferase; HSP, heat shock protein; Pro, proline; GB, glycine betaine; ABA, abscisic acid; PAs, polyamines; MAPK, mitogen-activated protein kinase; DREB, dehydration responsive element binding factor.

It is thus emerging that desaturation of membrane lipids, activation of reactive species (RS) scavengers, induction of molecular chaperones, and accumulation of compatible solutes are more generalized and conserved cellular defense responses. This is in line with the fact that membrane injury, RS damage, protein denaturation, and osmotic stress (primarily dehydration) can be provoked by a multitude of abiotic stresses. In stress response, these defenses are orchestrated by a complex regulatory network involving upstream signaling molecules including stress hormones [e.g., abscisic acid (ABA)], reactive oxygen species (ROS), hydrogen sulfide (H_2_S), nitric oxide (NO), polyamines (PAs), phytochromes, and calcium (Ca^2+^), as well as downstream gene regulation factors, particularly transcription factors (TFs) (Figure 1).

In the field, plants are routinely exposed to an unpredictable combination of different stresses rather than a single one (Slama et al., 2015; Wania et al., 2016), which is even worse in the context of climate change, soil salinization and environmental pollution. Particularly, on demand of population growth, it is getting imperative to equip crops with multistress tolerance. To this end, in this review, we are attempting to present an overview of the general defense systems and the major nodes of their underlying regulatory network (Figure 1), with an eye to their practical potential via genetic engineering and/or exogenous application.

## General Defenses Against Abiotic Stresses

In this section, five general botanic defenses against abiotic stresses will be addressed, comprising cuticle as outermost shield, unsaturated fatty acids (UFAs) as membrane modulator and oxylipin precursor, RS scavengers that govern RS homeostasis, molecular chaperones that stabilize proteins and subcellular structures (e.g., membrane), as well as compatible solutes that act more than osmoprotectants (Figure 1). Chances to acquire multistress tolerance based on them are given in Table 1. Notably, cuticular waxes, UFAs, antioxidant compounds, and compatible solutes are important economic traits as well. For example, waxes are raw materials of manifold products including biofuels, cosmetics, detergents, plastics, and pharmaceuticals (Lee and Suh, 2015). Therefore, increasing their yield actually serves a double purpose in crop improvement.

**Table 1:** Chances to generate multistress tolerance based on the general defenses.

General defenses	Methods
Cuticle	Overexpression of fatty acid condensing 3-ketoacyl-CoA synthase CER6
	Overexpression of alcohol-forming fatty acyl-CoA reductases (FARs)
	Overexpression of transporters, e.g., nonspecific lipid transfer proteins (nsLTPs)
Unsaturated fatty acids	Overexpression of ω-3 fatty acid desaturases (FAD3, FAD7, FAD8)
	Overexpression of lipid biosynthetic glycerol-3-phosphate acyltransferases (GPATs)
	Overexpression of enzyme cofactor acyl-carrier proteins (ACPs)
Reactive species scavengers	Overexpression of H_2_O_2_ reducing ascorbate peroxidases (APXs)
	Overexpression of GSH conjugating glutathione *S*-transferases (GSTs)
	Exogenous application of ROS scavenging cerium oxide nanoparticles
	Overexpression of methylglyoxal (MG) scavengers, e.g., the glyoxalase system
Molecular chaperones	Overexpression of heat shock proteins, e.g., HSP70, HSP16.4
Compatible solutes	Exogenous application of glycine betaine (GB)
	Antisense suppression of catabolic proline dehydrogenases (ProDHs)
	Exogenous application of proline (Pro)
	Overexpression of raffinose biosynthetic galactinol synthases (GOLSs)


### Cuticle

Land plants have an exterior translucent lipid structure, namely the cuticle, sealing the aerial surfaces of their organs. The thin hydrophobic layer is basically a cutin matrix filled with and coated by cuticular waxes. As the primary interface between plant and environment, the cuticle plays critical roles in restricting liquid and gas fluxes, defending pathogen and insect attacks, and resisting various abiotic stresses. It is an elegant innovation of land plants to deploy an outermost shield derived from simple molecules, which is fundamental to their success in terrestrial colonization (for review, see Shepherd and Wynne Griffiths, 2006; Yeats and Rose, 2013; Fich et al., 2016). By contrast, cell wall, the second barrier that is actively remodeled under abiotic stresses (Shen et al., 2014; Fernandes et al., 2016), is much more complex and less understood thus far (for review, see Le Gall et al., 2015; Tenhaken, 2016).

The cuticle is exclusively created by epidermal cells. Typically, cutin is a macromolecular polyester of C16 or C18 oxygenated fatty acids (FAs), whereas waxes are a complex mixture of C24 to C34 FA derivatives, including alcohols, aldehydes, alkanes, esters, and ketones. Their biosynthetic pathways are nearly resolved and have been well documented (see Pollard et al., 2008; Beisson et al., 2012; Fich et al., 2016 for cutin; see Shepherd and Wynne Griffiths, 2006; Kunst and Samuels, 2009; Bernard and Joubès, 2013 for waxes). Briefly, both of them stem from acetyl-coenzyme A (CoA) via *de novo* FA synthesis in plastids, with the accession of two carbons in each recurring cycle until the emergence of C16/C18 products, which are then transported to the endoplasmic reticulum (ER) to undergo either oxidation and incorporation to become cutin precursors (monoacylglycerols) or elongation and modifications to become wax components. Notably, two distinct modification pathways are involved in wax generation, the alcohol-forming (or acyl-reduction) pathway for primary alcohols and esters, together with the alkane-forming (or decarbonylation) pathway for aldehydes, alkanes, secondary alcohols, and ketones.

To assemble the apoplastic cuticle, these materials need to be exported from the ER to the plasma membrane (PM), and then across the PM through the cell wall onto the outer surface where cutin monomers polymerize and wax members crystallize. Membrane vesicle trafficking (McFarlane et al., 2014) is one of the ways involved in intracellular cargo delivery to the ATP-binding cassette (ABC) transporters that channel the PM (Pighin et al., 2004; Yeats and Rose, 2013; Fich et al., 2016). The likely extracellular relays for traversing the hydrophilic cell wall are non-specific lipid transfer proteins (nsLTPs), a group of small and basic proteins bearing a hydrophobic pocket for lipid binding. Indeed, two glycosylphosphatidylinositol-anchored LTPs, LTPG1 and LTPG2, as well as a secreted one, TsnsLTP4 from *Thellungiella*, have been reported to be implicated in wax deposition (Debono et al., 2009; Kim et al., 2012; Sun et al., 2015).

Then comes the last procedure of cutin production, i.e., the esterification of the monomers into a polymeric matrix. The crosslink is formed directly or via a bridging molecule, e.g., glycerol or ferulic acid (Deng et al., 2015; Fich et al., 2016). However, both the polymerization mechanism and the polyester architecture have been longstanding enigmas. The former is now beginning to be unveiled with the identification of CUTIN SYNTHASE 1 (CUS1), a cuticle-localized member of the GDSL lipase/hydrolase superfamily, from tomato (*Solanum lycopersicum*) (Girard et al., 2012; Yeats et al., 2012). Nevertheless, since the fruit of CUS1 null mutant, *cutin deficient 1* (*cd1*), are not fully deprived of cutin, non-enzymatic mechanisms cannot be ruled out yet (Yeats et al., 2012; Fich et al., 2016).

Drought tolerance is closely associated with wax accumulation in a wide variety of plant species (see reviews Borisjuk et al., 2014; Xue et al., 2017). With respect to multistress tolerance, however, wax composition makes a difference and the alcohol-forming pathway seems to outperform the alkane-forming one. ECERIFERUM 1 (CER1), the aldehyde decarbonylase (AD) responsible for *n*-alkane synthesis, could be reduced by cold, though induced by NaCl, mannitol (dehydration), and ABA in *Arabidopsis* (Bourdenx et al., 2011). It is highly possible that in CER1-overexpressing plants, cold tolerance was compromised, in parallel with pathogen defense and leaf growth, although water deficit resistance was improved (Bourdenx et al., 2011). Indeed, increased level of *n*-alkane coupled with decreased level of primary alcohols led to cold susceptibility and growth retardation. In contrast, higher contents of both resulted in better viability under drought and freezing without disturbing plant growth (Zhang et al., 2007). Besides, fatty acyl-CoA reductases (FARs) that produce primary alcohols could be up-regulated by cold, heat, polyethylene glycol (PEG; dehydration), ABA, methyl jasmonate (MeJA), and fungal infection in wheat (*Triticum aestivum*) (Chai et al., 2018). The stress-resistant performance of FAR-overexpressing plants is thus intriguing.

Another good candidate for genetic engineering might be CER6, the major 3-ketoacyl-CoA synthase (KCS) that catalyzes the initial and rate-limiting condensation step of FA elongation, as its overexpression could elevate total wax output with little alteration of the composition. Of note, it was not the cauliflower mosaic virus (CaMV) 35S promoter but the native one that could drive *CER6* expression high enough to achieve significantly greater wax quantity in transgenic *Arabidopsis* (Hooker et al., 2002). In addition to the enzymes, transporters can also be taken into consideration. Actually, *TsnsLTP4*, responsive to cold, heat, NaCl, PEG, and ABA, has been introduced into *Arabidopsis* and augmented its tolerance to drought and salt (Sun et al., 2015). Notably, more chances reside in manipulating the TFs that control cuticle generation, which will be discussed in the end.

### Unsaturated Fatty Acids

C16/C18 FAs are not only the prime stocks for the cuticle, but the key ingredients of the membranes, the fundamental biological barriers. The main building blocks of botanic membranes are phospholipids and glycolipids that both contain a glycerol core linked with two FA-derived “tails”. FAs thereby have a profound impact on membrane properties. Particularly, their unsaturation degree is a major determinant of membrane fluidity in that UFA chain will create a kink at a *cis*-double bond, which serves as steric hindrance in intermolecular package leading to a more fluid state (Hazel, 1995; Mikami and Murata, 2003).

Membrane fluidity is susceptible to various abiotic stresses, extreme temperatures in particular. Both cold-driven rigidification and heat-driven fluidization can cause biomembrane dysfunction, as exemplified by protein deactivation and ion leakage (Hazel, 1995). Cytoskeleton destabilization is also a direct consequence (Sangwan et al., 2002; Liu et al., 2013). Membrane remodeling is thus of especial importance in plants, which are poikilothermic organisms. Indeed, adjusting the unsaturation degree of the FA tails in bilayer interior is favored by plants in offsetting thermal perturbations to maintain the optimal range of fluidity. Particularly, there is a very close relationship between chilling tolerance and the unsaturation level of chloroplastic phosphatidylglycerol (PG) (for review, see Nishida and Murata, 1996; Iba, 2002).

In thylakoid membranes that are biased toward glycolipids, PG is the only phospholipid species present. Actually, it is an indispensable component of the membrane-bound photosynthetic apparatus including Photosystem II (PSII) (Wada and Murata, 2007). PSII is vulnerable to photoinhibition, in which the D1 protein of the reaction center is bound to continuous photodamage followed by repair via proteolysis and synthesis (Takahashi and Murata, 2008; Liu et al., 2016). Desaturation of PG has been shown to protect PSII against cold-enhanced photoinhibition, which contributes to chilling tolerance (Moon et al., 1995). This is also applicable to other stresses that can intensify photoinhibition (Takahashi and Murata, 2008). Upon NaCl treatment, for instance, alleviated photoinhibition of PSII pertained to increased contents of UFAs in membrane lipids including PG (Sui et al., 2010; Sui and Han, 2014; Liu et al., 2017). Indeed, specifically elevating the unsaturation level of PG accelerated the turnover of the D1 protein (Sun et al., 2010).

It is noteworthy that polyunsaturated UFAs, upon liberation by lipase from glycerolipids, also serve as the raw material of oxylipins, bioactive molecules involved in diverse physiological processes, including stress resistance (see review Savchenko et al., 2014). Particularly, linolenic acid (18:3) gives birth to jasmonic acid and its derivatives, namely jasmonates (JAs), a group of stress hormones with a well-understood role in launching wound response. There is emerging evidence that JA is also implicated in defense against other stresses, such as salt (Ryu and Cho, 2015; Yang et al., 2017) and UV (Mackerness et al., 1999; Conconi et al., 2006). It is of economic interest that applying MeJA to fruits and vegetables can reduce chilling injury, which is conducive to the maintenance of their post-harvest quality (González-Aguilar et al., 2000; Ding et al., 2002).

Unsaturation is administered by position-specific FA desaturases (FADs). To synthesize C18 UFAs that are more active in stress tolerance, C18 product of *de novo* FA synthesis linked to acyl-carrier protein (ACP), namely 18:0-ACP, is first converted to 18:1(9)-ACP by stearoyl-ACP desaturase (SAD). After being incorporated into glycerolipids, 18:1(9) is processed to 18:2(9, 12) by ω-6 desaturases and then to 18:3(9, 12, 15) by ω-3 desaturases (Murata and Los, 1997). To resist various stresses like cold and wounding, the level of 18:3 is usually elevated. This is much achieved by activation of ω-3 desaturases comprising ER-associated FAD3 and plastid-localized FAD7 and FAD8, as revealed by mounting evidence (Shi Y. et al., 2018; Sui et al., 2018). On the contrary, gene silencing of *FAD7* enabled transgenic tobacco (*Nicotiana tabacum*) to abide high temperatures (Murakami et al., 2000). Hence, inducible overexpression of FADs might be better so that heat tolerance can be covered.

The unsaturation level of chloroplast PG is otherwise determined by the substrate specificity of plastid glycerol-3-phosphate acyltransferase (GPAT), which catalyzes the first reaction to esterify FAs into glycerolipids. A preference for 18:1-ACP matters as the second reaction always utilizes 16:0-ACP (Nishida and Murata, 1996; Iba, 2002). Interestingly, GPATs from *Arabidopsis* (resistant) and squash (*Cucurbita moschata*) (sensitive) respectively assimilated the chilling behavior of tobacco (intermediate) (Murata et al., 1992), whereas GPAT from *Suaeda salsa* (euhalophyte) ameliorated salt tolerance of *Arabidopsis* (glycophyte) (Sui et al., 2017). Actually, under saline situations, *S. salsa* also exhibited chilling resistance (Cheng et al., 2014). Moreover, ACPs, as essential cofactors for FA synthase (FAS), SAD and GPAT, are also implicated in the alteration of FA composition. Transformation with *AhACP1* from peanut (*Arachis hypogaea*) into tobacco resulted in significantly higher contents of 18:2 and 18:3 accompanied with more tolerance against cold (Tang et al., 2012).

### Reactive Species Scavengers

An inherent paradox in normal metabolism of aerobic organisms is the endless generation of noxious RS, particularly ROS including superoxide anion (O_2_^•-^), hydrogen peroxide (H_2_O_2_), hydroxyl radical (^•^OH), and singlet oxygen (^1^O_2_), as well as reactive carbonyl species (RCS) like malondialdehyde [MDA; CH_2_(CHO)_2_] and methylglyoxal (MG; CH_3_COCHO). The two types of RS are intertwined with each other. RCS can arise from ROS-induced lipid peroxidation, while ROS can be raised by RCS activities the other way round. Virtually all abiotic stresses can trigger a burst of both ROS and RCS, turning their scavengers into general defenses. Nevertheless, ROS and MG have been identified to play a signaling role at low levels, which are also tactically exploited to facilitate stress perception and retort their elicitors [see review (Hasanuzzaman et al., 2017) for both ROS and MG]. Therefore, it is pivotal to maintain the delicate RS homeostasis, which needs to be taken into account in manipulating RS scavengers for multistress tolerance.

#### Reactive Oxygen Species

Plant cells carry an even heavier burden of ROS imposed by the electron transport chain of chloroplasts. Once overproduced, these small chemicals can readily attack various biomolecules encompassing carbohydrates, lipids, proteins, and nucleic acids, leading to oxidative catastrophe including enhanced photoinhibition and membrane lesions, which can be measured by the production of MDA from UFA peroxidation (Takahashi and Murata, 2008; Guo et al., 2012; Sui, 2015). Actually, MDA is a latent RCS that can initiate a new round of attack in acidic conditions, forming covalent adducts known as advanced lipoxidation end-products (ALEs), leading to protein dysfunction and consequent ROS proliferation (Farmer and Davoine, 2007; Deng et al., 2010).

Plants have therefore developed a sophisticated ROS scavenging system utilizing both non-enzymatic and enzymatic means. A good many metabolites possess antioxidant properties, such as betalains, carotenoids, flavonoids, and vitamin E (Gechev et al., 2006; Zhao S.Z. et al., 2011). Specialized enzymes comprise superoxide dismutases (SODs), catalases (CATs), and various peroxidases (PODs). SODs convert O_2_^•-^ into H_2_O_2_ for further reduction to water by CATs and PODs. Besides, the ascorbate-glutathione (ASA-GSH) cycle required for ascorbate peroxidase (APX) involves dehydroascorbate reductase (DHAR), monodehydroascorbate reductase (MDHAR) and glutathione reductase (GR). Other enzymes, such as glutathione *S*-transferase (GST) and ferritins, also partake in detoxification (see reviews Mittler et al., 2004; Gechev et al., 2006; Sharma et al., 2012).

Undoubtedly, multistress tolerance can be acquired via engineering the detoxifying enzymes. APXs, key enzymes ensuring H_2_O_2_ removal, helped transgenic plants oppose drought, salt and high light (Pang et al., 2011; Li K. et al., 2012; Cao et al., 2017), while GSTs, which catalyze GSH conjugation and bear glutathione peroxidase (GPX) activity as well, appended cold, heat, paraquat, UV, and heavy metals to the list (Roxas et al., 2000; Qi et al., 2010a; Kumar and Trivedi, 2018). However, exogenous application of cerium oxide nanoparticles (nanoceria) is emerging to be a more facile alternative, owing to their distinct capacities to catalytically clear ROS without substrate restriction and then readily regenerate via switch between the two oxidative states (Ce^3+^ and Ce^4+^) (Newkirk et al., 2018). One successful case is that anionic nanoceria with a low Ce^3+^/Ce^4+^ ratio applied to *Arabidopsis* leaves protected the photosynthetic machinery from chilling, heat and high light (Wu et al., 2017). Additionally, it should be cautious in crop cultivation that excessive use of fertilizer nitrogen can depress the ROS scavenging system leading to increased stress susceptibility (Kong et al., 2017).

#### Reactive Carbonyl Species

Methylglyoxal, a major type of RCS, is drawing increasing attention in stress scenario. In plant cells, glycolysis operates as the principal source of this cytotoxin, due to the non-enzymatic dephosphorylation of two intermediates, glyceraldehyde-3-phosphate and dihydroxyacetone phosphate. Once overaccumulated, MG can also damage various biomolecules, especially with its aldehyde group. In addition to forming advanced glycation end-products (AGEs) in analogy to ALEs, MG can further ROS production by catalyzing the photoreduction of O_2_ to O_2_^•-^ in chloroplasts and consuming GSH via spontaneous combination into hemithioacetal, leading to a vicious cycle and ultimate cell death (for review, see Hoque et al., 2016; Mostofa et al., 2018).

To detoxify MG and other 2-oxoaldehydes, plants have been armed with the glyoxalase system consisting of Gly I, Gly II, and Gly III. The former two enzymes work sequentially in a GSH-dependent way. The hemithioacetal adduct from MG and GSH is isomerized by Gly I into *S*-D-lactoylglutathione, which is then hydrolyzed by Gly II into D-lactate with the regeneration of GSH. By contrast, MG is directly converted to D-lactate by Gly III without the assistance of GSH, rendering a shortcut for its detoxification. Subsequently, D-lactate is processed into pyruvate by D-lactate dehydrogenase. It is ingenious that the toxic byproduct is not just eliminated, but recycled into an essential metabolite (Hoque et al., 2016; Hasanuzzaman et al., 2017; Mostofa et al., 2018). Remarkably, GSH not only serves as a bridge between the antioxidant and glyoxalase systems, but can also trap NO (see below), the primary reactive nitrogen species (RNS) interwoven with ROS, highlighting its significance in RS homeostasis and stress defense.

Genetic manipulation of the glyoxalase system, individually or together, to potentiate tolerance against multiple abiotic stresses has worked in various plant species, as exemplified by tobacco plants transformed with Gly I, which are capable of resisting drought, salt, heavy metals, and oxidative stress (Hoque et al., 2016). There are also some minor routes available for MG neutralization. For instance, MG, as with MDA, can be reduced by aldo-keto reductases (AKRs). Accordingly, ectopic expression of AKR guarded transgenic tobacco exposed to heat and oxidative stress (Hasanuzzaman et al., 2017). Interestingly, γ-aminobutyric acid (GABA), a natural amino acid with versatile roles including anti-stress signaling (see reviews Kinnersley, 2000; Bouché and Fromm, 2004), can trap MDA via direct reaction with the aldehyde groups (Deng et al., 2010), which means that MG can be blocked likewise.

### Molecular Chaperones

Heat shock proteins (HSPs) are well-known molecular chaperones, which are induced or constitutively expressed to facilitate protein folding, assembly, transport, and degradation. The anti-stress role of HSPs is not limited to their definition. In fact, this large family is a universal salvation system employed by virtually all living organisms to counteract all detrimental conditions that can induce protein damage, wherein they function to prevent aggregation of denatured proteins, assist in their refolding or present them to lysosomes or proteasomes for proteolysis, thereby restoring cellular homeostasis (for review, see Kregel, 2002; Wang et al., 2004). Besides, some unusually hydrophilic proteins, such as late embryogenesis abundant (LEA) and cold-regulated (COR) members might also function as chaperones to stabilize proteins and membranes against stress injury (see review Thomashow, 1999).

According to the molecular weight, there are five conserved HSP classes, namely HSP100/Clp, HSP90, HSP70/DnaK, HSP60/Chaperonin, and small HSP (smHSP). HSP70 is the most conserved one across different species, which consists of an N-terminal ATPase domain and a C-terminal substrate-binding domain. Binding and release of hydrophobic peptides rely on hydrolysis and recycling of ATP, which require the assistance of its co-chaperones including J-domain proteins (HSP40/DnaJ) that stimulate ATPase activity, and nucleotide exchange factors (NEFs) (e.g., HSPBP-1) that promote release of ADP and binding of fresh ATP. Intriguingly, *Arabidopsis* Fes1A, an ortholog of HSPBP-1, did not show NEF activity *in vitro*, but acted as an antagonist of HSP70 degradation (Zhang J.X. et al., 2010; Fu et al., 2015).

Notably, via physical interaction, HSP70 can modulate the activities of signal transducers, TFs, and/or metabolic enzymes, thereby exerting a profound influence on signaling pathways. Despite that it seems to be a negative regulator of HS response, substantial evidence has bonded it to thermoprotection in living organisms (Sung and Guy, 2003; Montero-Barrientos et al., 2010). Interestingly, higher SOD and POD activities even without HS were also brought to tobacco by *HSP70* from *Brassica campestris* (Wang X. et al., 2016), implying cross-tolerance to other stresses. However, it should be noted that in *Arabidopsis* its constitutive overproduction had pleiotropic consequences, dwarfism for instance (Sung and Guy, 2003).

By contrast, smHSP is the most diverse one in higher plants, with many subclasses distinct in protein sequence, cellular location and induction pattern, highlighting their special importance. smHSPs are clustered by the conserved C-terminal domain shared with vertebrate α-crystallin found in the eye lens, which is involved in oligomer formation and ATP-independent chaperone activity. Under stress challenges, drastically accumulated smHSPs are likely to seize non-native proteins to avoid their aggregation and then transfer them to ATP-dependent chaperones such as the HSP70 system for renaturation (see reviews Sun et al., 2002; Wang et al., 2004). It was newly reported that *Arabidopsis* transformed with *HSP16.4* from pepper (*Capsicum annuum*) were less vulnerable to drought, heat and their combination, with the ROS scavenging enzymes being more active under stressful conditions (Huang et al., 2018).

### Compatible Solutes

Compatible solutes are small organic compounds with electrical neutrality, high solubility and low toxicity that can even mount up to fairly high concentrations inside cells with few perturbations. Basically, qualified molecules are sugars, amino acids and their derivatives such as raffinose, trehalose, inositol, mannitol, proline (Pro), and glycine betaine (GB). In general, under stressful circumstances, these metabolites may accrue to act as osmoprotectants against dehydration, scavengers of RS, and/or stabilizers of proteins and membranes. Pro, a widely present one, is also able to buffer cellular redox potential and induce gene expression (for review, see Yancey, 2005; Ashraf and Foolad, 2007; Slama et al., 2015).

There have been successful cases of improving stress tolerance via genetic manipulating the metabolic enzymes of some compatible solutes. For example, raffinose is derived from sucrose via addition of galactose, which is donated by galactinol created from UDP-galactose and *myo*-inositol by galactinol synthase (GOLS). Transgenic plants expressing this key enzyme could withstand cold, drought and salt (Sun et al., 2013; Zhuo et al., 2013). Remarkably, antisense suppression of proline dehydrogenase (ProDH), which implements the first degradation step of Pro, even allowed *Arabidopsis* to survive -7°C and 600 mM NaCl (Nanjo et al., 1999). Alternatively, in spite that further knowledge is required to optimize the efficiency and minimize side effects, exogenous application of these solutes is emerging to be a more feasible and effective way, especially for GB, as the productivity of its biosynthetic enzymes was limited by substrate availability in engineered plants (Ashraf and Foolad, 2007).

## Regulatory Network Underlying Defense Systems

**FIGURE 2:**
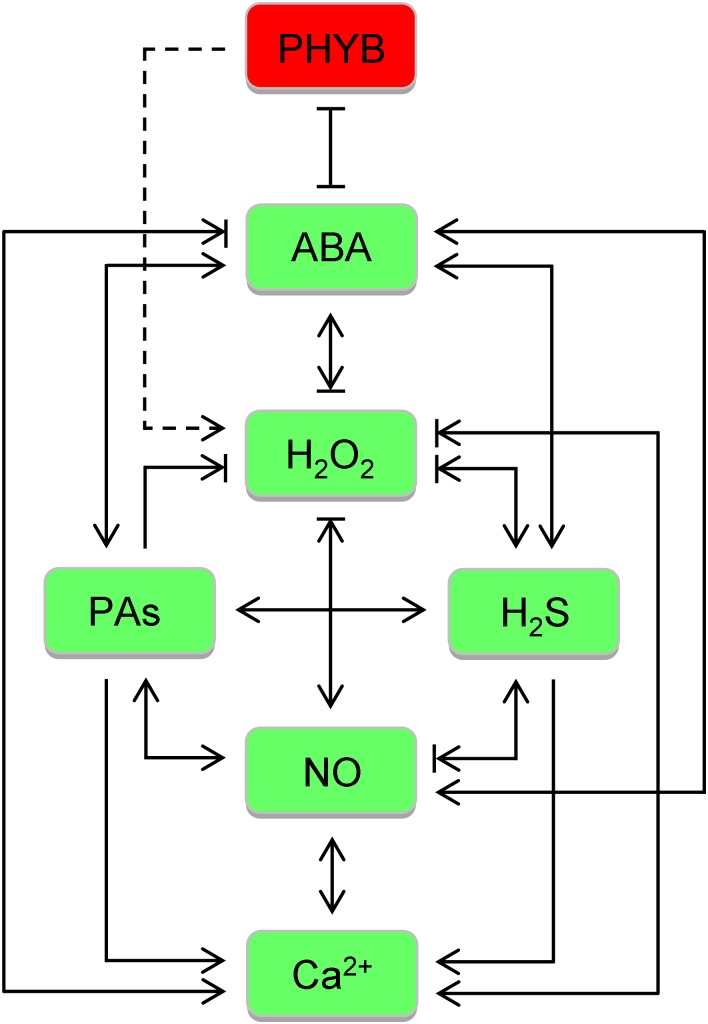
Crosstalk between signaling molecules focused in the review in botanic responses to abiotic stresses. Once triggered, abscisic acid (ABA), H_2_O_2_, H_2_S, NO, polyamines (PAs), phytochrome B (PHYB), and Ca^2+^, extensively interplay with others at various levels, synergistically or antagonistically. For simplification, the two effects are shown in combination. Dashed line is used between PHYB and H_2_O_2_ as PHYB is emerging to play a negative role in its scavenging. Of note, H_2_O_2_, H_2_S, NO, and PAs can actually block each other via chemical reaction, though not indicated.

In combat against abiotic stresses, the five general defenses are orchestrated by an intricate regulatory network composed of numerous signaling molecules and gene regulation factors. Here, we will just deal with some better characterized ones. Once triggered, stress hormones (ABA), ROS (H_2_O_2_), H_2_S, NO, PAs, phytochrome B (PHYB), and Ca^2+^, extensively interplay with others at various levels, synergistically or antagonistically (Figure 2), to establish a precise directive for downstream effectors, TFs in particular, to alter gene expression and protein/enzyme activities in a specific pattern, thereby launching a proper response (Figure 1). The regulatory factors render considerable opportunities to generate multistress tolerance, with examples related to the focused ones being listed in Table 2.

**Table 2:** Chances to generate multistress tolerance based on the regulatory network underlying the general defenses.

Regulatory factors	Methods
Abscisic acid (ABA)	Overexpression of biosynthetic enzyme 9-*cis*-epoxycarotenoiddioxygenase (NCED)
	Constitutive activation of receptors, e.g., pyrabactin resistance-like (PYL)
	Exogenous application of more stable analogs
H_2_O_2_	Overexpression of the MAPK pathway associated factors, e.g., ANP1, NDPK2, MKK2
NO	Exogenous application of chemical donors, e.g., GSNO, SNP
H_2_S	Exogenous application of chemical donors, e.g.,NaHS, GYY4137
Polyamines	Overexpression of biosynthetic enzymes, e.g.,arginine decarboxylase (ADC)
	Exogenous application
Phytochrome B (PHYB)	Deficient mutation
	Low R/FR ratio treatment
Ca^2+^	Overexpression of positive signaling components (CaMs, CBLs, CIPKs, CDPKs)
Transcription factors	Overexpression of activators, e.g., HSFs, DREB2C, MYB96, WXP1


### Stress Hormones

Phytohormones such as ABA, ethylene (ET), JA, and salicylic acid (SA) are important organizers of systemic stress defense, which coordinate in the elaborate hormonal signalsome (for review, see Wania et al., 2016; Tiwari et al., 2017). Notably, melatonin, a universal multi-regulatory molecule across all lifeforms, is increasingly recognized as a potent biostimulator against stress in plant. One notable aspect of this yet to be licensed phytohormone is that it operates as if a commander of other phytohormones (see reviews Arnao and Hernández-Ruiz, 2018; Kanwar et al., 2018). Nevertheless, ABA is the most prominent stress hormone, which not only extensively interplays with other phytohormones (see Table 3 for some updates), but with all following signaling molecules (Figure 2). Particularly, components of all biochemical defenses remarked above can be mobilized by ABA, including cuticular waxes (Lee and Suh, 2015), 18:3 (Yin et al., 2018), HSPs (Huang et al., 2016), Pro (Ashraf and Foolad, 2007), antioxidants (Liu F. et al., 2018), and RS detoxifying enzymes (Chen et al., 2013; Hoque et al., 2016).

**Table 3:** Updates on crosstalk between abscisic acid and other phytohormones.

Phytohormones	Crosstalk with abscisic acid (ABA)^a^	Reference
Auxin (IAA)	IbARF5 (Auxin response TF) from *Ipomoea batatas* can activate ABA biosynthetic genes in *Arabidopsis thaliana*	Kang et al., 2018
Brassinosteroid (BR)	ABA can activate BR signaling by reducing peu-miR-n68 to relieve BAK1 kinase in root growth of *Populus euphratica*	Lian et al., 2018
	ABA can inhibit BR signaling by promoting phosphorylation of BIN2 kinase in *A. thaliana*	Wang H. et al., 2018
Cytokinin (CK)	ABA can antagonize CK by repressing the expression of type-A ARRs in seed germination of *A. thaliana*	Huang et al., 2017
Ethylene (ET)	CaHSFB2a (TF) is responsive to ABA, ET, JA, and SA in *Capsicum annuum*	Ashraf et al., 2018
	FvWRKY42 (TF) is responsive to ABA, ET, JA, and SA in *Fragaria vesca*	Wei et al., 2018
Gibberellin (GA)	ABA can activate GA signaling by inducing peu-miR477 to repress RGL1 (GA repressor) in root growth of *P. euphratica*	Lian et al., 2018
	ABF3 (TF) may be a converging point of ABA, GA and JA pathways in drought response of *A. thaliana*	Liu S. et al., 2018
Jasmonate (JA)	GmSK1 (E3 ubiquitin ligase component) is responsive to ABA, JA and SA in *Glycine max*	Chen et al., 2018
	MYC2 (TF) can interact with ABF3 to connect JA and ABA pathways in drought response of *A. thaliana*	Liu S. et al., 2018
Salicylic acid (SA)	ABA and H_2_O_2_ mediate SA-induced freezing tolerance of *Triticum aestivum*	Wang W. et al., 2018
	PCaP2 (Ca^2+^-binding protein) is shared by ABA and SA pathways in water deficit tolerance of *A. thaliana*	Wang X. et al., 2018
Strigolactone (SL)	ABA (via H_2_O_2_) can induce SL production to improve salt tolerance of arbuscular mycorrhizal *Sesbania cannabina*	Ren et al., 2018


*^a^TF, transcription factor; ARRs, *Arabidopsis* response regulators.*

Stress stimuli can rapidly trigger *de novo* synthesis of ABA from oxidative cleavage of β-carotene, with 9-*cis*-epoxycarotenoiddioxygenase (NCED) being the rate-limiting enzyme. For sequestration of this hormone, two ways are available. One is hydroxylation involving cytochrome P450 type enzyme (CYP707A), the other is conjugation to glucose by glycosyltransferase. The latter actually creates a bin for ABA recycling carried out by glycosidase, offering a shortcut for stress induction. To establish systemic response, ABA can be actively transported across the PM by ABC transporters and then recognized by pyrabactin resistance (PYR), PYR-like (PYL) or regulatory component of ABA receptors (RCAR) proteins. Coupled with ABA, PYR/PYL/RCAR binds to and inhibits protein phosphatase 2Cs (PP2Cs), allowing activation of the sucrose non-fermenting 1-related protein kinase 2 (SnRK2) family members. Upon phosphorylation, downstream targets, such as ion channels, metabolic enzymes and TFs, effectuate robust stress response. Besides, the mitogen-activated protein kinase (MAPK) pathway is also involved in ABA signaling (for review, see Sreenivasulu et al., 2012; Ng et al., 2014; Sah et al., 2016).

Significantly, ABA can also induce organic changes to cope with unfavorable situations. The well-known one is the closure of stomata, minute pores formed by paired guard cells to permit gas exchange, which can reduce water loss from transpiration and thus mitigate dehydration. This movement is achieved by modulating the activities of ion channels and aquaporins. As a result, outflow of K^+^ and anions drags water out by osmosis, leading to guard cell shrinkage, which is ensured by corresponding rearrangement of actin filaments (Zhao Y. et al., 2011; Zhao et al., 2016). Another special one is the dormancy of seed, which can avoid the existing stresses and await conditions suitable for germination. The viability of stressed seed is largely dependent on their coat properties (Xu et al., 2016; Song et al., 2017b) and cotyledon chlorophyll content (Zhang S.R. et al., 2010; Li X. et al., 2012).

Both endogenous elevation and exogenous addition of ABA are efficient in supporting plants confronting with various stresses. The development of ABA analogs with higher stability is promising in field application. However, overloaded ABA signaling, as in the case of overexpressed NCED or constitutively active PYLs, may also lead to vegetative growth retardation and grain yield reduction (Sreenivasulu et al., 2012; Ng et al., 2014), while foliar spraying of ABA can induce leaf senescence, as observed in rice (*Oryza sativa*) and maize (*Zea mays*) (Sah et al., 2016). Therefore, it is important to get better understandings of ABA homeostasis, its extensive biological effects and crosstalk with other pathways for designing strategies that can impart crop stress tolerance at little expense of the economic traits.

### Reactive Oxygen Species

*In planta*, ROS are continuously generated as byproducts of aerobic metabolism in distinct intracellular compartments involving chloroplasts, mitochondria, and peroxisomes. However, as already mentioned, they are not just toxins that need to be removed, but signaling molecules indispensable for diverse physiological processes including stress resistance. Of note, an ROS signal is shaped by multiple factors such as dose, duration, origin, and type (Gechev et al., 2006). Herein, the focus is on H_2_O_2_, the relatively stable and less reactive ROS that takes the core node of stress signaling. The extensive crosstalk between H_2_O_2_ and other signaling molecules, including ABA, ET, JA, SA, NO, and Ca^2+^, has been reviewed in (Saxena et al., 2016). Particularly, Ca^2+^ influx is a notable event in H_2_O_2_ signaling, which, in turn, modulates the level of H_2_O_2_ via activating the producing (e.g., RBOHs below) or scavenging enzymes. Notably, H_2_O_2_ is intrinsically tied to PAs as a product of their degradation (Liu et al., 2007). The more complicated interactions between H_2_O_2_ and two gasotransmitters (H_2_S and NO) will be discussed later on.

In the apoplast, ROS can be initiatively produced upon stress stimuli by various enzymes. The major ones are respiratory burst oxidase homologs (RBOHs), the PM-localized NADPH oxidases that are activated by binding of Ca^2+^ to the EF-hand motifs in the N-terminal cytosolic region, with the synergy of phosphorylation by, for instance, receptor-like cytoplasmic protein kinases (RLCKs). In *Arabidopsis*, two RBOH isoforms, RBOHD and RBOHF, are employed to yield ROS against both abiotic and biotic stresses. Particularly, they are essential in ROS-dependent ABA signaling such as stomatal closure and seed dormancy. Their product is O_2_^•-^, which then gives rise to H_2_O_2_ via both spontaneous and SOD-catalyzed dismutation. The low abundance of apoplastic ASA and GSH, the two major redox buffers, allows propagation of H_2_O_2_ to be perceived extracellularly by yet unclear sensors and/or imported via aquaporins (though freely diffusible) into cells to turn on intracellular signaling, thereby launching rapid local or systemic response (see reviews Jalmi and Sinha, 2015; Kimura et al., 2017).

In intracellular signaling of H_2_O_2_ originating from metabolic disturbance and/or apoplastic release, the MAPK signalsome plays a central role. Actually, it is an important converging node of stress signaling. Each phosphorylation cascade is constituted by three kinases, MAPK kinase kinase (MAPKKK), MAPK kinase (MAPKK), and MAPK. To date, many MAPKs and different cascades have been identified to differentially decode H_2_O_2_ signal, albeit it remains elusive how the specificity is determined. Inversely, the MAPK pathway is also functioning upstream of ROS via modulating, positively or negatively, the activities of RBOHs. In *Arabidopsis*, two MAPKs, MPK3 and MPK6, are positioned in defensive response, which can ultimately elevate the levels of defensive factors like GSTs and HSPs. H_2_O_2_ can prompt them not only via ANP1, a MAPKKK, but via other kinases, including oxidative signal-inducible 1 (OXI1) that is necessary for full activation of MPK3/6, and NUCLEOTIDE DIPHOSPHATE KINASE 2 (NDPK2) that can interact with, and potentiate the activities of, MPK3/6. As expected, NPK1 (tobacco ANP1 ortholog) and AtNDPK2 both granted transgenic plants tolerance to multiple stresses, including salt and extreme temperatures, so did MKK2, a MAPKK in another anti-stress cascade, namely MEKK1-MKK2-MPK4/6 (see reviews Jalmi and Sinha, 2015; Liu and He, 2017).

Besides, H_2_O_2_ may also ensure MAPK activation by inhibiting their repressors like protein tyrosine phosphatases (PTPs) via oxidizing the thiol (-SH) group of the cysteine residue (Jalmi and Sinha, 2015). Actually, reversible thiol oxidation may be a direct and important way in conveying H_2_O_2_ signal, rendering a large pool of substrates as potential sensors (see reviews Choudhury et al., 2017; Kimura et al., 2017). With the consequently altered functions of diverse effectors, including kinases, phosphatases, TFs, metabolic enzymes, and ion channels, cellular processes are extensively rearranged. Interestingly, thiol modification may also offer a node for signal crosstalk and modulation via raising a competition between H_2_O_2_ and other factors, including the two gasotransmitters (see below).

### Hydrogen Sulfide and Nitric Oxide

As with H_2_O_2_, two toxic gaseous molecules, H_2_S and NO, at low concentrations, also display impressive powers in safeguarding plants against a broad spectrum of stresses. The two share in common many anti-stress mechanisms. For example, both can alleviate salt toxicity via activating SALT OVERLY SENSITIVE 1 (SOS1), a PM Na^+^/H^+^ antiporter, to increase Na^+^ exclusion (Deng et al., 2016; Kong et al., 2016). The most conspicuous role should be their ability to squash oxidative stress, wherein both of them not only act as antioxidants in their own right but can repress ROS production and activate ROS elimination. Particularly, as a source of sulfur, H_2_S can be assimilated into GSH, leading to a boost in this essential RS scavenger (see reviews Siddiqui et al., 2011; Hancock and Whiteman, 2014). Not surprisingly, carbon monoxide (CO), the first recognized gasotransmitter, is also an elicitor of stress response, though CO research in this theme is still in its infancy (see review Wang and Liao, 2016).

The three RS, H_2_O_2_, H_2_S, and NO, are usually present together during various stresses and exhibit intricate interactions depending on the context. For example, in stomata regulation, H_2_O_2_ and NO synergistically mediate ABA-induced closure, whereas H_2_S acts as a Janus. With the antagonist face, it can ablate NO accumulation and abet stomata opening (see review Lisjak et al., 2013). However, NO is a mediator of H_2_S in promoting adventitious root formation (Zhang et al., 2009), which can increase the uptake of O_2_ and thus attenuate hypoxia stress from waterlogging (Song et al., 2011; Chen et al., 2016). On the contrary, H_2_S is a mediator of NO in heat tolerance of maize (Li Z.G. et al., 2013) and in cadmium resistance of bermudagrass (*Cynodon dactylon*) (Shi et al., 2014). Noteworthily, this reverse demonstrates that H_2_S is not a referee that functions through monitoring ROS and NO, as once proposed (Hancock and Whiteman, 2014), but indeed an active player, albeit the three are not always in the same team. Moreover, with the emergence of another RS player, namely MG, the situation will be further complicated.

Their chemical reactions add one more layer of complexity, which can block each other yet bring about new compounds with potential physiological effects, such as peroxynitrite (ONOO-) formed by NO and O_2_^•-^, as well as nitrosothiols formed by NO and H_2_S. Interestingly, as already mentioned, there is even a competition among them, with the participance of MG and GSH, since all of them can directly modulate protein function via thiol modification, namely, oxidation by H_2_O_2_, sulfhydration by H_2_S, nitrosylation by NO, glycation by MG, and glutathionylation by GSH (Lisjak et al., 2013; Mostofa et al., 2018). Actually, GSH *per se* offers an additional way for their crosstalk, as it is a derivative of H_2_S, but a quencher of the other three. Besides, the MAPK pathway is likely to be a convergent point of the four signaling RS.

Being notorious as air pollutants, the two gasotransmitters are actually natural products of botanic metabolism from diverse origins. Enzymatic examples include cysteine desulfhydrases (DES) and sulfite reductase (SIR) for H_2_S, as well as nitrate reductase (NR) and the nitric oxide synthase (NOS)-like way for NO, though a real NOS has as yet not been identified *in planta*. With respect to their removal, *O*-acetylserine(thiol)lyase (OAS-TL) can consume H_2_S in cysteine production, while being trapped by GSH in the conjugate *S*-nitrosoglutathione (GSNO) provides a way for NO storage, transport and degradation, which will be deaminated by GSNO reductase (GSNOR) into glutathione disulfide (GSSG) and NH_3_ (see reviews Calderwood and Kopriva, 2014; Farnese et al., 2016).

For practical use, however, both of them are readily supplied by chemical donors, such as GSNO and sodium nitroprusside (SNP) for NO, as well as sodium hydrosulfide (NaHS) and GYY4137 for H_2_S. Notably, GYY4137 is a phosphorodithioate derivative that can release H_2_S slowly and steadily under physiological conditions. A pile of literature has substantially proved that foliar spray of these donors is a highly effective approach to aid plants in combating manifold stresses (see reviews Guo H. et al., 2016; Fancy et al., 2017).

### Polyamines

Polyamines are a group of organic compounds with aliphatic nitrogen structure. The diamine putrescine (Put), triamine spermidine (Spd), and tetraamine spermine (Spm) are natural PAs shared by almost all living organisms, which are short-chain polycations derived from arginine/ornithine via clear pathways (see Liu et al., 2007; Gill and Tuteja, 2010). The protective role of PAs in plant response to a wide range of stresses has long been recognized (see reviews Liu et al., 2007; Alcázar et al., 2010; Gill and Tuteja, 2010; Minocha et al., 2014). Indeed, overexpression of every PA biosynthetic enzyme, such as arginine decarboxylase (ADC), spermidine synthase (SPDS) and *S*-adenosylmethionine synthetase (SAMS), advanced stress tolerance in various plant species, so did exogenous application of PAs (Alcázar et al., 2010; Qi et al., 2010b; Minocha et al., 2014).

A complexity arises in dissecting the mechanisms underlying the anti-stress effects of PAs. It is plausible that these multi-faceted substances contribute to stress defense in diverse ways, owing to their polycationic nature, RS-scavenging property, and signaling function. For example, at the physiological PH, protonated PAs not only participate in ion homeostasis *per se*, but can bind to negatively charged molecules including membrane lipids and integral proteins, which help mitigate stress-induced membrane damage. In stress signaling, PAs not only communicate with ABA, but can induce rapid production of NO. Peculiarly, PAs are affiliated with other metabolites involved in stress response, including Pro and ET interconnected with PA anabolism, as well as H_2_O_2_ and GABA generated from PA catabolism (see Alcázar et al., 2010; Minocha et al., 2014). Of note, due to the special link with H_2_O_2_, PAs also fall into the Janus category (see review Gupta et al., 2016), which need to be taken into consideration in their practical application.

### Phytochromes

PHYB is emerging as a negative regulator in stress tolerance, which belongs to a small family of chromophore-containing proteins that serve as photoreceptors to perceive red (R) and far-red (FR) light. The signaling activity of PHYB is subjected to reversible photoconversion composed of R activation and FR deactivation based on conformational change. Nascent PHYB is in the inactive Pr (R-absorbing) form. Once converted to the bioactive Pfr (FR-absorbing) form, dimeric PHYB will translocate into the nucleus, where it can interact with, and trigger the proteasomal degradation of, phytochrome interacting factors (PIFs), a subfamily of basic helix-loop-helix (bHLH) TFs, so as to remodel the expression profile of thousands of light-responsive genes, thereby guiding photomorphogenesis (Franklin and Quail, 2010; Zhou et al., 2014).

More recently, PHYB was identified to be a thermosensor as well (Jung et al., 2016; Legris et al., 2016). Warm ambient temperatures can effectively induce elongation growth, which phenocopies shade avoidance controlled by the PHY-PIF cascade. Indeed, Pfr can also revert to Pr in a spontaneous way called thermal (or dark) reversion, which is independent of light but sensitive to temperature. Therefore, warm temperatures, particularly during night, can relieve the repression of PIF4 via quickly deactivating PHYB, together with enhancing the transcription of PIF4, thereby driving thermomorphogenesis. As active PHYB was found to interact with PIF-binding sites (G-boxes) at PIF4-targeted promoters, it was proposed to have an additional inhibitory role as a co-repressor or competitor of PIF4 in gene regulation (see review Delker et al., 2017). Another cascade downstream of PHYB in light- and temperature-induced growth involves the RING E3 ligase CONSTITUTIVE PHOTOMORPHOGENIC 1 (COP1) and the TF ELONGATED HYPOCOTYL 5 (HY5), with COP1 ubiquitinating HY5 for degradation to derepress the growth genes. Notably, COP1 can indirectly potentiate the activity of PIF4, thereby connecting the two branches (see review Legris et al., 2017).

Loss-of-function of PHYB upon mutation or low R/FR ratio treatment can ameliorate cold tolerance in that active PHYB indirectly represses the expression of TFs belonging to the C-REPEAT BINDING FACTOR/DEHYDRATION RESPONSIVE ELEMENT BINDING FACTOR 1 (CBF/DREB1) family, which play a central role in cold acclimation via activating downstream targets such as the *COR* genes (Franklin and Whitelam, 2007; He et al., 2016; Wang F. et al., 2016). The direct preys of PHYB are activators of the CBF/DREB1 regulon, such as the ABA-dependent JA signaling in tomato (Wang F. et al., 2016) and the PIF-like protein OsPIL16 in rice (He et al., 2016). Notably, in rice *phyB* mutant, UFA content is much higher than in wild type (WT), leading to better chloroplast structure and less photoinhibition under chilling stress (Yang et al., 2013). Interestingly, stronger heat tolerance has been observed in *Arabidopsis phyB* mutant, with the HS damper of lateral root development being relieved (Song et al., 2017a). This might be related to the reduction of HY5, which is a negative regulator of the unfolded protein response (UPR) that can be triggered by HS (Nawkar et al., 2017).

Rice *phyB* mutant also exhibits better drought tolerance owing to decreased transpiration rate involving two morphological changes. One is reduced total leaf area per plant, which is probably due to inhibited leaf cell proliferation. The other is reduced stomatal density, which results from enhanced epidermal cell expansion by activation of putative ERECTA family and EXPANSIN family genes (Liu et al., 2012). However, in contrast to the observations in *Arabidopsis* (Boccalandro et al., 2009; Casson et al., 2009), stomatal development is not affected in rice by *phyB* deficiency, albeit reduced stomatal density is the common outcome. Besides, PHYB is the mediator of R light-induced stomatal opening (Wang et al., 2010), which might be related to its repression effect on PIFs as well, as maize ZmPIF1 was found to contribute in ABA-induced stomatal closure (Gao et al., 2018). Interestingly, it was later noted in rice *phyB* mutant that the expression levels and enzymatic activities of APX and CAT were significantly higher than those in WT when deprived of water, reflecting a negative role of PHYB in ROS scavenging (Yoo et al., 2017).

Similarly, antioxidant enzymes were more active in tobacco *phyB* mutant, which was newly reported to be more tolerant to salt (Yang et al., 2018). NtPHYB could suppress the biosynthesis of ABA and JA via targeting the enzyme genes, as has been suggested for aforementioned tomato SlPHYB (Wang F. et al., 2016). Inversely, ABA cooperated with JA to inhibit the expression of *NtPHYB*, in consistence with another new finding that ABA significantly reduced the transcription of *OsPHYB* for drought escape (Du et al., 2018). It seems that such mutual regulation is common to various stress responses. Moreover, PHYA, the only light-labile family member, antagonized PHYB in regulating chilling signaling of tomato (Wang F. et al., 2016); however, it synergized, and even surpassed, PHYB in attenuating salinity response of tobacco (Yang et al., 2018).

### Calcium Ion

Ca^2+^, a versatile secondary messenger, is involved in plant responses to virtually all abiotic stresses, directly or indirectly via other signaling molecules, serving as a key integration node of the regulatory network. Upon stimulation, Ca^2+^ is mobilized from the reservoirs, apoplast and vacuole in particular. The signaling is ignited by sharp influx through Ca^2+^ channels, such as the PM cyclic nucleotide-gated channels (CNGCs) and the tonoplast TWO PORE CHANNEL 1 (TPC1) (Lu et al., 2016; Zheng et al., 2017), and soon quenched by efflux via Ca^2+^-ATPases and Ca^2+^/H^+^ exchangers (Han et al., 2011, 2012). Notably, the latter, as well as other secondary transporters, are energized by virtue of the proton gradient established by PM H^+^-ATPase, vacuolar H^+^-ATPase (V-ATPase) and vacuolar H^+^-translocating inorganic pyrophosphatase (V-PPase). These H^+^ pumps are thus pivotal in ionic and osmotic homeostasis, thereby contributing to salt tolerance in particular (Chen M. et al., 2010; Yang et al., 2010; Yuan et al., 2016b).

Influent Ca^2+^ is perceived by various Ca^2+^ sensors, including calmodulins (CaMs), calcineurin B-like proteins (CBLs), and Ca^2+^-dependent protein kinases (CDPKs) that contain EF-hand Ca^2+^-binding motifs. Unlike CDPKs that are immediate effectors, CaMs and CBLs usually need to evoke downstream effectors. CaMs can interact with a large variety of targets, such as kinases, phosphatases and TFs, whereas CBLs primarily bind to CBL-interacting protein kinases (CIPKs). With gene expression and protein/enzyme activities being altered by different effectors, positively or negatively, Ca^2+^ signatures are deciphered into specific cellular responses. Besides ABA biosynthetic enzymes, a good example is glutamate decarboxylase (GAD), which is rapidly activated by binding of Ca^2+^-CaM to convert L-glutamate into GABA, leading to accumulation of this multifunctional anti-stress molecule (for review, see Luan et al., 2002; Shi S. et al., 2018). NO production can also be promoted by CaMs, but via inhibiting the degradation enzyme GSNOR under saline conditions (Zhou S. et al., 2016).

Moreover, as a cation antagonistic to Na^+^, Ca^2+^ is intrinsically implicated in salt detoxification with modulating ion transporters being an important means. To maintain a properly high K^+^/Na^+^ ratio in the cytosol that is critical in salt tolerance (Chen et al., 2005; Feng et al., 2015), Na^+^ scarcity is accomplished by activating the Na^+^/H^+^ antiporter SOS1 via the SOS3-SOS2 (CBL4-CIPK24) cascade to increase Na^+^ efflux, together with blocking non-selective cation channels (NSCCs) to decrease Na^+^ influx (Luan et al., 2002; Ding et al., 2010). Meanwhile, K^+^ equilibrium is monitored by CBLs through adjusting, directly or indirectly, the activity of the inward rectifier ARABIDOPSIS K^+^ TRANSPORTER 1 (AKT1) (Ren et al., 2013). Furthermore, Ca^2+^ itself is necessary for stabilizing cell wall and membranes (Shi S. et al., 2018).

It is thus not surprising that overexpression of CaMs (Zhou S. et al., 2016), CBLs (Cheong et al., 2003; Cheong et al., 2010), CIPKs (Pan et al., 2018), or CDPKs (Xu et al., 2010; Asano et al., 2012), even exogenous supplement of CaCl_2_ (Ahmad et al., 2018), all enabled plants to bear salt toxicity. Remarkably, transgenic rice harboring CIPKs from wild barley (*Hordeum spontaneum*) displayed enhanced tolerance to drought and heavy metals as well. Besides, some *HsCIPKs* were also subject to induction by cold, heat and ABA (Pan et al., 2018). However, the behavior of *Arabidopsis cbl1* mutant upon cold exposure was really inconsequent, with higher (Cheong et al., 2003), similar (Albrecht et al., 2003), and lower (Huang et al., 2011) tolerance all being reported. Taking into account that plants were correspondingly cultured under light, dark, and 16 h light/8 h dark during the treatment, as well as the opposite roles of PHYA and PHYB in cold response, one possibility is that CBL1 might be involved in signaling of both phytochromes, with different CIPKs being evoked.

### Gene Regulation Factors

Once the transduced stress cue is received, gene regulation factors active at different levels, including histone acetyltransferases (HATs) (Stockinger et al., 2001), TFs, alternative splicing factors (Laloum et al., 2018), microRNAs (Zhang et al., 2013), and ubiquitination enzymes (Lee and Suh, 2015) are engaged in fine-tuning the defense systems. Transcriptional level is still the key regulatory node. Scores of TFs have been identified to be stress-responsive, such as ABA-responsive element (ABRE) binding proteins/factors (AREBs/ABFs) (Sah et al., 2016), DELLAs (An et al., 2015), NACs (Tang et al., 2017), WRKYs (Bai et al., 2018), zinc finger proteins (Han et al., 2014; Wang K. et al., 2018), and the APETALA2/ETHYLENE RESPONSE FACTOR (AP2/ERF) superfamily, to which the aforementioned CBF/DREB1 family belongs (Mizoi et al., 2012).

Notably, heat shock factors (HSFs) not only serve as the master regulator of HSPs, but can monitor their own members and many other defense factors, including APX and GST that eliminate ROS, as well as GOLS essential for raffinose synthesis, via binding upon oligomerization to the heat shock elements (HSEs) located in their promoter regions. Therefore, HSFs are actually capable of launching three general defensive systems. Strikingly, the number of HSFs in plants is large and highly variable, 16 in peanut (*A. duranensis*) vs. 52 in soybean (*Glycine max*) for example (Scharf et al., 2012; Wang P.F. et al., 2017), not to mention their functional diversification. Such multiplicity perplexes their study and application. Besides, negative effects have been observed in ectopic expression of HSFs. A case is that tomato SlHSFA3 increased seed germination sensitivity to salt in *Arabidopsis* (Li Z. et al., 2013). Nonetheless, genetic manipulation of HSFs is still a promising avenue to confer plants multiplex tolerance (for review, see Scharf et al., 2012; Guo M. et al., 2016). Moreover, it is worth mentioning that HSFA6b and HSFA3 enable ABA to be a *bona fide* participant of HS response. The former is directly activated by AREB1, while the latter is downstream of DREB2A, a target shared by AREB1 and HSFA6b (Huang et al., 2016).

DREB2C, another class 2 DREB member, is an activator of HSFA3 as well (Chen H. et al., 2010; Hwang et al., 2012). It can also target potential chaperones like COR15A and DESICCATION-RESPONSIVE PROTEIN 29A (RD29A) in mitigating salt toxicity (Song et al., 2014). Interestingly, DREB2C from *Ammopiptanthus mongolicus*, a evergreen broadleaf shrub living in desert, was newly reported to up-regulate not only the three factors, but Δ1-pyrroline-5-carboxylate synthetase (P5CS) that initiates Pro biosynthesis, as well as FADs that catalyze 18:3 production, thereby promoting *Arabidopsis* endurance to drought, freezing and heat (Yin et al., 2018). A safe conclusion can then be drawn that this single TF governs all of the four cellular general defenses. Besides, computational analysis conducted on *Arabidopsis DREB2C* promoter has identified diverse types of *cis*-acting elements, which are responsive to ABA (ABRE), MeJA, SA (TCA), heat (HSE), low temperature (LTR), and stress (TC rich), respectively (Sazegari et al., 2015), suggesting that this TF is a core converging point in stress signaling. Indeed, it is an ABA-inducible TF and can exert a positive feedback on ABA biosynthesis via *trans*-activating *NCED9* to delay seed germination (Je et al., 2014).

To finalize, light is shed back on transcriptional regulation of cuticle biosynthesis. In *Arabidopsis*, the enzyme genes are controlled primarily by TFs of two families, including SHINE 1 (SHN1), -2, -3, and DEWAX of the AP2/ERF superfamily, as well as MYB16, MYB30 and MYB106 of the R2R3-MYB family. Interestingly, MYB96 is an activator of wax production, whereas MYB41 is a repressor of cutin synthesis (see reviews Borisjuk et al., 2014; Lee and Suh, 2015). Notably, MYB96 is a prominent implementer of ABA signaling, with the whole wax metabolism being put under control. Not only elongation and modification enzymes, but ABC transporters and nsLTPs have at least one isoform gene targeted, directly or indirectly (Seo et al., 2011). MYB96 transgenesis upgraded drought and freezing tolerance of *Arabidopsis*; however, significant dwarfism was a concomitant (Seo et al., 2009; Guo et al., 2013). By contrast, wax production 1 (WXP1), an ERF member from *Medicago truncatula*, might be a better candidate, which was the one accounting for the previously mentioned observation that higher contents of both *n*-alkane and primary alcohols resulted in better viability under drought and freezing without disturbing the growth of transgenic *Arabidopsis* (Zhang et al., 2007).

## Conclusion and Perspectives

As one of the successful habitants thriving on the earth, there is no great surprise that plants have found smart ways to deal with abiotic stresses. The existence of general defense systems raises the feasibility to endow crops and other plants with multistress tolerance in a simplified way, albeit there are still many gaps need to be filled in before their field application. To avoid the pleiotropic effects from over-activation, as observed for ABA and HSFs, synthetic stress-inducible promoters (Hou et al., 2012) could be a method of choice. It is worth trying to find an optimal cocktail of the defensive molecules that can balance each other to minimize undesired effects. Of note, NO may be able to antagonize ABA in leaf senescence (Kong et al., 2016). Screening for mutants, generated from gamma irradiation (Yuan et al., 2015a) for instance, that are stress-resilient but overcome side effects is pretty sound. Moreover, defense genes from stress-resistant species that might have acquired adaptive function, as in the case of *GPAT*, are good candidates for transgenesis.

With the help of high-throughput techniques and bioinformatic platforms, we will be able to learn more from the natural existing extremophiles like *Thellungiella*, and acquire more comprehensive and in-depth understandings of the stress responses of different crops. It would be more informative to challenge them with a combination of stresses that mimics to some extent the field conditions. Interestingly, the earth has actually been offering silicon (Si) as “compensation”. This abundant element in the crust plays significant roles in the easing of both abiotic and biotic stresses, though the mechanisms are under debating (see reviews Debona et al., 2017; Coskun et al., 2018). With a better knowledge of Si utilization, benefits from the addition of Si in fertilizers can be envisaged. Furthermore, plant species that have the capacity to deprive soils of salt and heavy metals, such as *S. salsa* (Song and Wang, 2015), are highly instrumental in the restoration of arable land for sustainable agriculture.

## Author Contributions

All authors listed have made a substantial, direct and intellectual contribution to the work, and approved it for publication.

## Conflict of Interest Statement

The authors declare that the research was conducted in the absence of any commercial or financial relationships that could be construed as a potential conflict of interest.
